# Multi-state models for the analysis of time-to-treatment modification among HIV patients under highly active antiretroviral therapy in Southwest Ethiopia

**DOI:** 10.1186/s12879-017-2533-3

**Published:** 2017-06-27

**Authors:** Belay Birlie, Roel Braekers, Tadesse Awoke, Adetayo Kasim, Ziv Shkedy

**Affiliations:** 1 Department of Statistics, Jimma University, Jimma, Ethiopia; 2 I-BioStat, Hasselt University, Diepenbeek, Belgium; 3 Institute of public Health, University of Gondar, Gondar, Ethiopia; 4 Wolfson Research Institute for Health and Wellbeing, Durham University, Manchester, UK

**Keywords:** HIV/AIDS, Highly active antiretroviral therapy, Treatment modification, Survial analysis, Multistate models

## Abstract

**Background:**

Highly active antiretroviral therapy (HAART) has shown a dramatic change in controlling the burden of HIV/AIDS. However, the new challenge of HAART is to allow long-term sustainability. Toxicities, comorbidity, pregnancy, and treatment failure, among others, would result in frequent initial HAART regimen change. The aim of this study was to evaluate the durability of first line antiretroviral therapy and to assess the causes of initial highly active antiretroviral therapeutic regimen changes among patients on HAART.

**Methods:**

A Hospital based retrospective study was conducted from January 2007 to August 2013 at Jimma University Hospital, Southwest Ethiopia. Data on the prescribed ARV along with start date, switching date, and reason for change was collected. The primary outcome was defined as the time-to-treatment change. We adopted a multi-state survival modeling approach assuming each treatment regimen as *state*. We estimate the transition probability of patients to move from one regimen to another.

**Result:**

A total of 1284 ART naive patients were included in the study. Almost half of the patients (41.2%) changed their treatment during follow up for various reasons; 442 (34.4%) changed once and 86 (6.69%) changed more than once. Toxicity was the most common reason for treatment changes accounting for 48.94% of the changes, followed by comorbidity (New TB) 14.31%. The HAART combinations that were robust to treatment changes were *tenofovir (TDF) + lamivudine (3TC)+ efavirenz (EFV), tenofovir + lamivudine (3TC) + nevirapine (NVP)* and *zidovudine (AZT) + lamivudine (3TC) + nevirapine (NVP)* with 3.6%, 4.5% and 11% treatment changes, respectively.

**Conclusion:**

Moving away from drugs with poor safety profiles, such as *stavudine*(d4T), could reduce modification rates and this would improve regimen tolerability, while preserving future treatment options.

**Electronic supplementary material:**

The online version of this article (doi:10.1186/s12879-017-2533-3) contains supplementary material, which is available to authorized users.

## Background

The implementation of HAART at a large scale has shown a dramatic change in controlling the burden of HIV/AIDS. Various studies from developed as well as developing countries have reported an improvement in CD4 cell counts following ART initiation [1–6] and decreases in mortality [7].

Currently, Ethiopia and most resource-limited countries have adopted non-nucleoside reverse transcriptase inhibitors (NNRTIs) based therapy. They use either NVP or EFV plus two nucleoside reverse transcriptase inhibitors (NRTI) as a first line therapy for adults and adolescents. This combination has been shown to be efficacious, are generally less expensive, and have generic formulations [7]. However, the new challenge of HAART is to allow long-term durability. Many patients will be forced to modify or switch their treatment regimens for various reasons, including poor drug tolerance, drug toxicities, drug-to-drug interactions, pregnancy and treatment failure [8–10].

Studies from developed and developing countries have shown that a substantial number of patients (up to 69%) may modify their regimen overtime, where 25% - 44% of them modify their initial treatment within the first years of treatment [8–14]. Drug related toxicity was the most common reason for treatment modification [8, 9, 11, 13, 15–18] and this can be an important barrier to adherence and potentially lead to treatment failure [19]. The majority of these studies found that patients that receive d4T as a part of their treatment were at increased risk of treatment modification due to toxicity [13, 15–18], which raises questions about the continued role of d4T in first-line treatment. The WHO had revised its guidelines on the use of antiretroviral drugs several times. Recently, WHO recommends to move away from d4T giving preference to the use of TDF and AZT in standard first-line therapy when possible [20, 21]. Due to cost and management of toxicity, however, the transition from d4T to TDF has been slow in resource-limited settings [20, 21].

The high rate of HAART switching emphasizes the complexity of managing these therapies. Given the limited number of second-line treatment options available in resource-limited settings, maximizing regimen durability by minimizing the rate of treatment modification and rates of treatment failure amongst those on first-line regimens is vital to extend first-line treatment options and prevent premature initiation of second-line therapy. In order to achieve this goal, key reasons for changes in ART regimens should be studied and durable regimens should be identified for recommendations. Moreover, evaluating the influence of initial ART regimens on the likelihood of treatment modification has a vital role in determining what treatment to initiate and what treatment to preserve. Although relevant data on patients’ long-term experience on ART from resource limited settings are less commonly available, some investigators have described the reasons for modification of HAART and compared durability of individual ARV’s using routine clinical programme data [15–18, 22–27]. However, the majority of these studies have had short follow-up times and consider only first time regimen switching or first time single drug substitution with no distinction made between NNRTI and NRTI substitutions.

Therefore, this study aims to compare the durability of first-line ART regimens and investigate reasons for treatment modification in patients under HAART in Jimma university specialized hospital. For this purpose, we adopted a multi-state survival modeling approach assuming each treatment regimen as *state*. We estimate the transition probability of patients to move from one regimen to another in general as well as due to a specific event that triggers the move. The proposed model allows modelling of both the occurrence of different event types (such as, single drug substitution or regimen switch) and the occurrence of subsequent events, the latter potentially of different types.

## Methods

### Data

#### Description of the cohort

The data used for this study were obtained from Jimma University Specialized Hospital HIV/AIDS clinic, located 352 Km Southwest of Addis Ababa, Ethiopia. The Hospital gives VCT, PMTCT and free ART service for people living in Jimma Town and Southwest Ethiopia. Patients begin ART after they have been checked for medical eligibility and are counseled for adherence for ART. Patients presenting with WHO stage 4 disease and/or a CD4 count lower than 200 were eligible to start ART. Those who started ART, have a regular follow-up for drug adverse effects, management of opportunistic infection, TB screen and counseling related to family planning. In addition, CD4 count is measured at each visit. Viral load measurement is not available. Adverse event monitoring is conducted by clinicians during medical visits in accordance with national guidelines.

Decisions on which treatment regimen to start or substitute are made by the clinician in consultation with the patient. During the study period, the standard first-line regimens to be initiated were d4T + 3TC + NVP (1), d4T + 3TC + EFV (2), AZT + 3TC + NVP (3), AZT + 3TC + EFV (4), TDF + 3TC + EFV (5), and TDF + 3TC + NVP (6). If a patient suffered from side effects/toxicities related to the NRTI’s (d4T, AZT or TDF), and was not in need of second-line therapy for virologic failure, the recommendation was to substitute d4T with either AZT or TDF, to substitute AZT either with d4T or TDF, and to substitute TDF with either d4T or AZT. Similarly, patients initiated on the NNRTI EFV could substitute with NVP, while those on NVP could substitute with EFV. In addition, upon the recommendation of WHO, in Oct 2012 the hospital started to phase out d4T backbone by replacing either AZT or TDF for patients who were on d4T based regimen.All data, including demographic, clinical conditions, laboratory test results and medications are recorded and entered in to the database by a data entry clerk at the clinic. In addition, data on prescribed ARV along with start and stop dates of the drug and reasons for discontinuation are documented. Use of Jimma University Hospital HIV/AIDS clinic data and analysis of de-identified data was approved by the Human Research Ethics Committee of Jimma University.

#### Study population

All ART naive patients, aged 18 years or older and who initiated a standard, public-sector, first-line ART regimen at the clinic between between January 1, 2007 and December 31, 2011 were eligible for this analysis. The data was closed for analysis at the end of August, 2013.

#### Outcome

The primary outcome was defined as the time-to-treatment change (treatment modification or regimen switching). For the purpose of this study, treatment change is defined as changing at least one ARV in the regimen without initiating a second-line therapy. ARV dosage adjustments were not considered as treatment change. Time zero was defined as the day of ART initiation and each recurrent treatment change time was measured from the beginning of the patient’s ART initiation in months. Person-time of the study subject ended at the earliest of initiation on second-line therapy, lost to follow up, death, transfer or closure of the data set for analysis (August 25, 2013).

### Multi-state survival model

#### Model formulation

**Fig. 1:**
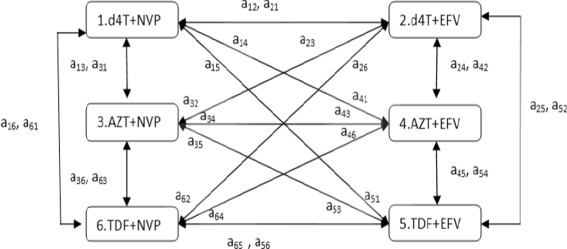
A Six-state multi state model for treatment change. Note that 3TC was omitted because it was present in all the regimens. The transition intensities matrix is presented in Additional file 1: Section S2

Possible transition between treatment combinations are presented in Fig. 1 which illustrates the treatment history of patients under ART. From here onward we use the term state to denote a specific treatment combination. The model has 6 transient states (which represents 6 first-line treatment combinations): d4T + 3TC + NVP (1), d4T + 3TC + EFV (2), AZT + 3TC + NVP (3), AZT + 3TC + EFV (4), TDF + 3TC + EFV (5), and TDF + 3TC + NVP (6). The model assumes that every patient can switch to all the regimen at one point or other. However, a patient can only switch to one regime at a time. For example, a patient who started treatment with d4T + 3TC + NVP State 1) is at risk of making one of the following transitions at a particular time; 1→2,1→3,1→4,1→5 and 1→6. If the patients made the transition 1→2 or 1→3, the subject has undergone a single drug-substitution (treatment modification). Transition 1→2 implies that the patient has substituted their NNRTI’s NVP by EFV without changing their NRTI treatment (d4T). However, transition 1→3 implies that the patient has substituted their NRTI’s d4T by AZT without changing their NNRTI treatment. Transitions 1→4 or 1→5, imply regimen switching, substituting both NNRTI and NRTI at the same time. After making one of these possible transitions patients will be at risk of making further transition.

Let (*X*_*t*_)_*t*>0_ be a multi-state process with a state space {1,2,…,6}. The stochastic process (*X*_*t*_)_*t*>0_ is defined as *X*_*t*_=*ℓ*, if the process is in state *ℓ* at time *t* (in months). As mentioned above, for the case study presented in this paper, there are 6 possible first lines regimens which implies that the initial state of the patient *X*_0_∈{1,2,…,6}.

Our main interest is to model the transition from *ℓ* ^*th*^ regimen (state *ℓ*) to *j* ^*th*^ regimen (state *j*) at time *t*. A transition will be simply denoted by *ℓ* *j*. The distribution of this multi-state process is characterized by the transition intensities, or hazard rate, *a*_*ℓj*_(*t*), which expresses the instantaneous risk of a transition from state *ℓ* into state *j* at time *t*, that is

1 $$ {}\begin{aligned} a_{\ell j}(t)={\lim}_{\Delta t \rightarrow 0} \frac{P(X_{(t+\Delta t)}=j|X_{t}=\ell,{F}_{t^{-}})}{\Delta t}, \ell, j \in \{1,2,...,6\}, \ell \neq j. \end{aligned}  $$

Here, ${F}_{t^{-}}\phantom {\dot {i}\!}$ represents process history prior to time *t*. In our application, time *t* represents time since ART initiation. The cumulative transition hazards is defined as $A_{\ell j}(t) = \int _{0}^{t} a_{\ell j}(u)du, u \leq t, \text {where}\ A_{\ell j}(t) = 0$ if a direct transition between state *ℓ* and *j* is impossible. These intensities can be gathered in to a 6×6 matrix **A**(*t*) with diagonal elements $A_{\ell \ell }(t)=-\sum _{\substack {j=1,j \neq \ell }}^{6} A_{\ell j}(t), \ell, j = \{1,2, \ldots, 6\}$. Note that individuals who have no transition should remain on their initial regimen (starting state) after ART initiation.

A central issue related to ART management is the ability to estimate the probability of the future treatment combination of the patient (i.e. the patient state) given all the information available until the present moment. For example, given a patient who substituted his NRTI’s d4T by AZT without changing his NNRTI after 6 months and (i.e., the current state of the patient is either in state 3 or state 4, depending on the initial NNRTI component) who has had no further events at one year post ART, one may be interested in estimating the probability of staying on this combination for additional 6 months as well as in comparing this probability to a patient who did not substitute their NRTI (d4T). We propose to use the transition probabilities for long-term prediction of a patient’s state. Let *s* be the time at which the prediction is made measured from the time origin of the patient (start of treatment) and let us denote the event history of the patient up to time *s* by *X*_*u*_,0≤*u*≤*s*. Then, the transition probability from state *ℓ* to state *j* in the time interval [*s*,*t*], given information available until time *s*, is defined as

2 $$ {}\begin{aligned} P_{\ell j}(s,t) = P(X_{t} = j \mid X_{s}=\ell,X_{u}), s \leq t, \ell,j \in \{1,2,...,6\}, u \in\, [0,s]\!. \end{aligned}  $$

In order to estimate *P*_*ℓj*_(*s*,*t*) we proposed to use a Markov model [28]. The model assumes that the future course of the patient depends on the patient’s state in the current time but not on the patient’s history before the current state. This means that, conditional on the present state, the past has no influence on the risk. This implies that

3 $$ {}\begin{aligned} a_{\ell j}(t)dt=P(X_{(t+\Delta t)^{-}}=j|X_{t^{-}}=\ell), \ell, j \in \{1,2,...,6\}, \ell \neq j \end{aligned}  $$

and

4 $$ {}\begin{aligned} P_{\ell j}(s,t) = P(X_{t} = j \mid X_{s}=\ell), s \leq t,\ell,j \in \{1,2,...,6\}. \end{aligned}  $$

Analogous to **A**(*t*),these probabilities can be gathered in to a 6×6 matrix **M**(*s*,*t*) with (*P*_*ℓj*_(*s*,*t*)) as its (*ℓ*,*j*)^*th*^ entry. A single element (*P*_*ℓj*_(*s*,*t*)) combines both direct and indirect transition from state *ℓ* to state *j*. The matrix is fully presented in Additional file 1: Section S.2.

#### Inference

In this section we present non-parametric approaches for time continuous Markov models with finite state space and under independent right censoring. We consider *n* individual multistate processes $\left (X^{(i)}_{t}\right)_{t \geq 0}, X^{(i)}_{t} \in \{1,...,6\},\ \text {and}\ i=1,2,...,n$. We assume that the *n* process are all, conditional on the initial state $X^{(i)}_{0}$, independent multistate processes. Observation of the individual multistate data is subject to a right censoring time *C*_*i*_. Our notation and ideas are based on a counting process formulation [29, 30].

Let *N*_*ℓj*;*i*_(*t*) be the counting process denoting individual *i* ^′^ *s* number of observed direct (without visiting another state in between) *ℓ*→*j* transition in [0,*t*],*ℓ*,*j*∈1,2,…,6,*ℓ*≠*j*. Here, time *t* refers to the time since the patient entered the initial state (i.e., the time since ART initiation). Let *Y*_*ℓ*;*i*_(*t*) be an indicator variable which represent the at risk process where we have

$${}{\begin{aligned} Y_{\ell;i}(t) = \left\{ \begin{array}{cl} 1 & : \text{if individual}~ i~ \text{is in state}~ \ell~ \text{and under observation before time}~ t,\\ 0 & :\text{otherwise.} \end{array}\right. \end{aligned}} $$

Let the aggregated process $N_{\ell j}(t) = \sum _{i=1}^{n} N_{\ell j;i} (t), \ell \neq j$ and $Y_{\ell }(t) = \sum _{i=1}^{n} Y_{\ell ;i}(t)$ denote, respectively, the number of observed direct *ℓ*→*j* transitions during the time interval [0,*t*] and the number of individuals to be observed at risk in state *ℓ* just prior to time *t*. We define the the increment of *N*_*ℓj*_(*t*) as △*N*_*ℓj*_(*t*)=*N*_*ℓj*_(*t*)−*N*_*ℓj*_(*t* ^−^) for the increment of *N*_*ℓj*_(*t*) which gives the number of *ℓ*→*j* transitions observed exactly at time *t*.

#### Nonparametric estimation of baseline hazards

From the definition of the transition intensities in Eq. (3) *a*_*ℓj*_ *d* *t*=*P*(*X*_(*t*+*dt*)−_=*j*∣*X*_*t*−_=*ℓ*),*ℓ*≠*j*. Hence, if we observe no *ℓ*→*j* transition at *t* (i.e △*N*_*ℓj*_(*t*)=0) we estimate the increment *a*_*ℓj*_(*t*)*d* *t* of the cumulative hazard as 0. If we do observe *ℓ*→*j* transition at *t* (i.e △*N*_*ℓj*_(*t*)>0), we estimate this conditional transition probability by

5 $$ \triangle \hat{A}_{\ell j}(t) = \frac{\triangle N_{\ell j}(t)}{Y_{\ell}(t)},  $$

summing up over these increments yields the Nelson-Aalen estimators [29]

6 $$ \hat{A}_{\ell j}(t) =\sum\limits_{s \leq t} \frac{\triangle N_{\ell j}(s)}{Y_{\ell} (s)}, \ell \neq j,  $$

where summation is over all observed event times in [0,*t*] and its variance is given by

7 $$ \hat{\delta}_{\ell j}^{2}(t) =\sum\limits_{s \leq t} \frac{\triangle N_{\ell j}(s)}{Y_{\ell}^{2}(s)}, \ell \neq j.  $$

#### Nonparametric estimation of Transition probabilities

The transition probabilities are a complex function of the transition hazards, because the state occupied at some time *t* may potentially result from a complex nested series of competing risks experiments and there may also be more than one possible sequence of competing risks experiments leading to being in a certain state at a certain time [31]. Under the Markov model the transition probabilities defined in (4) are the solution of a set of differential equations [29]

8 $$ \frac{d}{dt}\mathbf{M}(s,t) =\mathbf{A}^{T}(t)\mathbf{M}(s,t),  $$

where **M**(*s*,*t*) is transition probability matrix with (*ℓ*,*j*) element *P*_*ℓj*_(*s*,*t*)=*P*(*X*_*t*_=*j*∣*X*_*s*_=*ℓ*) and **A**(*t*) is a matrix with off diagonal elements *A*_*ℓj*_(*t*)=*a*_*ℓℓ*_(*t*) and diagonal elements $A_{\ell \ell }(t)=-\sum _{\substack {j=1,j \neq \ell }}^{6} a_{\ell j}(t)$. In coordinates, (8) is $d/dt P_{\ell j}(s,t) =\sum _{k} P_{\ell k}(s,t)A_{kj}(t)$ and for any fixed initial state *ℓ*, the vector of transition probabilities from *ℓ*, (*P*_*ℓ*1_(*s*,*t*),*P*_*ℓ*2_(*s*,*t*),...,*P*_*ℓ*6_(*s*,*t*)) satisfies this equation. Even though this equation can not be solved in general due to the non-constancy over time of the matrix **A**(*t*), under Markov assumption, the transition probabilities satisfy

9 $$ P_{\ell j}(s,t)=\sum_{r} P_{\ell r}(s,u)P_{rj}(u,t)\;\;, s \le u \le t,  $$

and based on a partition *s*=*t*_0_<*t*_1_<*t*_2_<…<*t*_*k*−1_<*t*_*k*_=*t* of the time interval [*s*,*t*], the matrix of transition probabilities **M**(*s*,*t*) can be approximated by [31]

10 $$ \mathbf{M}(s,t) \approx \prod\limits_{k=1}^{K} (\mathbf{I}+\triangle \mathbf{A}(t_{k})),  $$

where **I** is the (6×6) identity matrix, the (*ℓ*,*j*)^th^ element of △**A**(*t*_*k*_) is equal to *A*_*ℓj*_(*t*_*k*_)−*A*_*ℓj*_(*t*_*k*−1_), and $A_{\ell \ell }(t)=-\sum _{\substack {j=1,j \neq l }}^{6} A_{lj}(t)$. Computing the approximation for ever finer partition [*s*,*t*] approaches a limit, namely a matrix-valued product integral [31, 32], which equals the matrix of transition probabilities,

11 $$ \mathbf{M}(s,t)=\prod\limits_{u \in (s,t]}(\mathbf{I}+d\mathbf{A}(u)),  $$

where *u* ranges from *s* to *t* and *d* **A**(*u*) is defined as *d* *A*_*ℓj*_(*u*)=*a*_*ℓj*_(*u*)*d* *u*,*ℓ*,*j*∈1,2,...,6 [29]. Therefore, for Markov models, given **A**(*t*), the product integration is the mapping that switches from cumulative transition hazards to the matrix of transition probabilities while all cumulative transition hazards are involved.

An estimator of **M**(*s*,*t*) can be obtained by replacing **A**(*u*) with the Nelson-Aalen estimators, $\hat {\mathbf {A}}(u)$, and by defining $d\hat {\mathbf {A}}(u)$ as the matrix with entries $\triangle \hat {A}(u)= \hat {A}_{\ell j}(u) - \hat {A}_{\ell j}(u^{-})$ (i.e., the increment of the Nelson-Aalen estimators at time *u*). This results in the Aalen-Johansen type estimator [29],

12 $$ \hat{\mathbf{M}}(s,t) = \prod\limits_{\substack{u \in (s,t]}}(\mathbf{I} + \Delta \hat{\mathbf{A}}(u)),  $$

in which *u* indicates all event times in (*s*,*t*]. Note that the transition probability matrix defined in (11) is calculated by means of a product integral, while its estimator in (12) is based on a finite product, which only changes at event times.

The transition probabilities can be used for two types of prediction: forward and fixed horizon [30, 33]. In the former case, at a given fixed time *s* the probabilities of possible future events are evaluated for varying time horizons *t*. In the latter case, the prediction is made from several starting points to one future fixed time point. In both cases, Aalen-type or Greenwood type estimators of the variance-covariance matrix of **M**(*s*,*t*) can be calculated directly or through a recursion formula which can for instance be used to construct point-wise confidence intervals around the estimated transition probability curves [30]. In our application we use forward prediction type.

#### Robustness of First-line HAART towards Treatment Modification/change

The primary aim of this study is to quantify the robustness of first line treatments to treatment modification. Given the individuals initial state *ℓ* at time *s*, the waiting time in state *ℓ*. i.e., the duration of stay at state *ℓ*, can be used as a summary measure of the model. The waiting time in state *ℓ* is generated with hazard

$$a_{\ell.}(t)=\sum\limits_{j=1,j \neq \ell}^{6} a_{\ell j}(t), t>=0. $$

We define the total cumulative transition hazard out of state *ℓ* as $ A_{\ell.}(t)=\int _{0}^{t} a_{\ell.}(u)du=\sum _{j=1,j \neq \ell }^{6} A_{\ell j}(t)$. Using these quantities one can evaluate the probability of no events during a period. The survival function of the waiting time in the initial state *ℓ*, i.e., probability to stay in state *ℓ* until time *t*, given that the individual had already been in the respective state at time *s*,*s*≤*t* is given by,

$$\begin{array}{*{20}l} P(X_{t}=\ell|x_{s}=\ell) &= \prod\limits_{\substack{u \in (s,t]}}(1-a_{\ell.}(u)du)\\ &=exp \left(-\int_{s}^{t}a_{\ell.}(u)du\right)\\&=\text{exp}(-A_{\ell.}(t)) \ell=1,...,6. \end{array} $$

These quantities are essentially common survival probabilities with all cause hazard *a*_*ℓ*._(*u*), taking time *s* as time origin [31]. However, this can also be seen as a solution of the product integral in (11). Since, the *ℓ* ^*th*^ row of the Aalen-Johansen type estimator of $\hat {\textbf {M}}(s,t)$ contains the estimates $\hat {P}_{\ell j}(s, t)$ for *ℓ*≠*j* and the diagonal element is such that the sum over the *ℓ* ^*th*^ row equals 1, the Aalen-Johansen estimator of *P*(*X*_*t*_=*ℓ*|*x*_*s*_=*ℓ*) is just $\hat {P}_{\ell \ell }(s,t)$.

The multi-state model formulated above allows us to evaluate whether treatment modification reflect a substitution of NNRTI, substitution of NRTI or substitution of both NNRTI and NRTI by initial treatment combinations. We propose to use the following measures of HAART robustness to treatment modification:

1. Probability of NNRTI substitution
*P*_12_ for state 1 *P*_34_ for state 3 *P*_56_ for state 5
*P*_21_ for state 2 *P*_43_ for state 4 *P*_65_ for state 6
2. Probability of NRTI substitution
*P*_13_+*P*_16_ for state 1 *P*_35_+*P*_36_ for state 3 *P*_54_+*P*_52_ for state 5
*P*_24_+*P*_25_ for state 2 *P*_42_+*P*_45_ for state 4 *P*_63_+*P*_61_ for state 6
3. Probability of regimen switching
*P*_14_+*P*_15_ for state 1 *P*_32_+*P*_35_ for state 3 *P*_53_+*P*_51_ for state 5
*P*_23_+*P*_26_ for state 2 *P*_41_+*P*_46_ for state 4 *P*_64_+*P*_62_ for state 6

## Result

Of the 1453 eligible patients, 169 patients were excluded because of limited follow up (i.e those with at most 1 month follow-up data) and missing information (patients with missing information about prescribed ARV or start and stop dates of the drug). A total of 1284 subjects were included for the analysis presented in this paper. Patients person-time were cut at the earliest of changing to second line treatment, death, lost to followup, transfer or end of study (Aug 25, 2013). The median follow-up time was 37.40 months (IQR: 22.32-56.15 months) and the average follow-up time was 38.25 months per person. At ART initiation, patients had a median CD4 cell count of 137*c* *e* *l* *l* *s*/*m* *m* ^3^ (IQR: 78-201 *c* *e* *l* *l* *s*/*m* *m* ^3^), were predominately female (68.81%) and had a median age of 30 years (IQR: 26-35 years) (Table 1). The most common regimens initiated were d4T + 3TC + EFV consisting of 526(40.96%) patients while the treatment TDF + 3TC + EFV was administered to 401(31.23%) patients at initiation.

**Table 1:** Baseline characteristics of study subjects

Characteristics		n(%)
Gender (n(%))	Male	439(34.19)
	Female	845(68.81)
WHO Stage(n(%))	SI	372(28.97)
	SII	398(30.99)
	SIII	419(32.63)
	SIV	95(7.41)
Treatment at start(n(%))	1	526(40.96)
	2	67(5.22)
	3	185(14.41)
	4	82 (6.39)
	5	401(31.23)
	6	23(1.79)
Age (Median (IQR))		30 (26-35)
Baseline CD4 (Median (IQR))		137(78-201)
Status at the end(n(%))	Dead	52(4.05)
	Drop	197(15.34)
	Transfer	127(9.89)
	Under follow up	908(70.72)

Note: 1: d4T + 3TC + NVP, 2: d4T + 3TC + EFV, 3: AZT + 3TC + NVP, 4: AZT + 3TC + EFV, 5: TDF + 3TC + EFV, and 6: TDF + 3TC + NVP

For the majority of the patients (58.8%) the first line treatment was not modified, 442 patients (34.4%) had their treatment changed only once while 86 patients (6.69%) had their treatment changed more than once. In total, 615 (32.4%) treatment changes occurred in this cohort over the period of follow-up. Of those 615 changes, 426 (69.27%) substituted NRTI only, 144 (23.41%) substituted NNRTI only and 45 (7.32%) substituted both the NRTI and NNRTI at the same time. The number of events (transition made from each state) and the number of patients in total that were at risk for treatment modification are presented in Table 2. Five hundred forty one patients were on d4T + 3TC + NVP combination, but only 125 (23%) remained on this regimen without any modification. 89% of the 483 patients who were on AZT + 3TC + NVP and 95% of the 89 patients who were on TDF + 3TC + NVP did not experience treatment modification. Among the regimens containing EFV, 36% of 157 patients on d4T + 3TC + EFV, 86% of 161 patients on AZT + 3TC + EFV and 96% of 471 patients on TDF + 3TC + EFV did not experience treatment modification. The frequency of treatment change was the lowest amongst those patients initiated on TDF-based regimens (3.75%) compared to those initiated on AZT (11.96%) and d4T-based regimens (73.92%). It is interesting that regimens containing d4T were more prone to treatment modification than those containing AZT and TDF. Apart from d4T, patients who received NVP (42.5%) were more susceptible to treatment modification than patients who received EFV (17.87%).

**Table 2:** Observed transition matrix

	1	2	3	4	5	6	No event	Total entering
1	-	87(0.16)	271(0.50)	2(0.004)	15(0.028)	41(0.08)	125(0.23)	541
2	5(0.03)	-	12(0.08)	48(0.31)	34(0.22)	1(0.006)	57(0.36)	157
3	7(0.014)	0(0.00)	-	25(0.052)	10(0.021)	11(0.023)	430(0.89)	483
4	1(0.006)	1(0.006)	12(0.075)	-	10(0.063)	0(0.000)	137(0.85)	161
5	0(0.000)	1(0.002)	2(0.004)	1(0.002)	-	13(0.027)	454(0.96)	471
6	0(0.000)	0(0.000)	1(0.011)	1(0.011)	2(0.022)	-	85(0.95)	89

Note: 1: d4T + 3TC + NVP, 2: d4T + 3TC + EFV, 3: AZT + 3TC + NVP, 4: AZT + 3TC + EFV, 5: TDF + 3TC + EFV, and 6: TDF + 3TC + NVP. The *ℓ* *j* ^*th*^ entries are the frequencies (percentage) of transition from state *ℓ* to state *j*. Those in column "no event" are the numbers of patients ending the study in each state and those in column "total entering" are the numbers of patients observed to be in each state at some time point prior to the end of the study

**Fig. 2:**
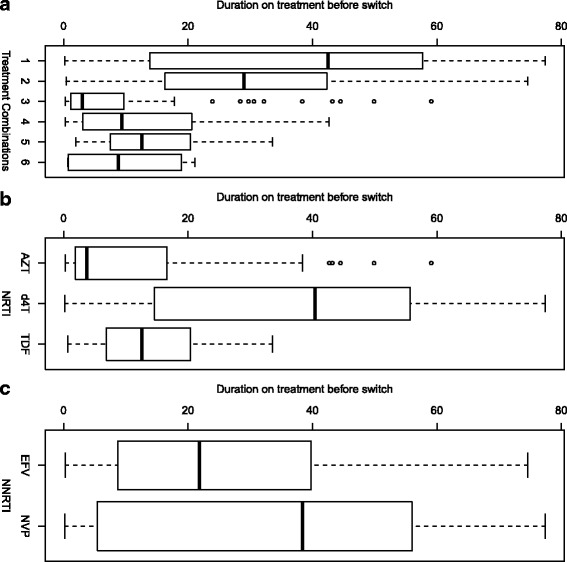
Duration on treatment before switch in months. **a** Duration in original treatment combination before switch, **b** Duration in original NRTI before switch, and **c** Duration in original NNRTI before switch. Note that only the time spent in the current treatment combination of the patients who modified their treatment are considered

As seen from Fig. 2 (Panel b), however, when we look at the time spent in the current treatment combination of the patients who modified their treatment, patients initiated on d4T had a tendency to stay longer (40.40 months; IQR: 14.60-55.73) as compared to AZT patients (3.7 months; IQR: 1.90-16.02) and TDF patients (12.60 months; IQR: 6.84-20.40). Similarly, patients initiated on NVP had a tendency to stay longer (38.37 months; IQR: 5.42-56.05) as compared to EFV patients (21.80 months; IQR: 8.70-39.80) (Fig. 2 (Panel c)). The duration of stay in each treatment combination before the first change to another treatment combination is presented in Additional file 1: Figure S3.1.

**Fig. 3:**
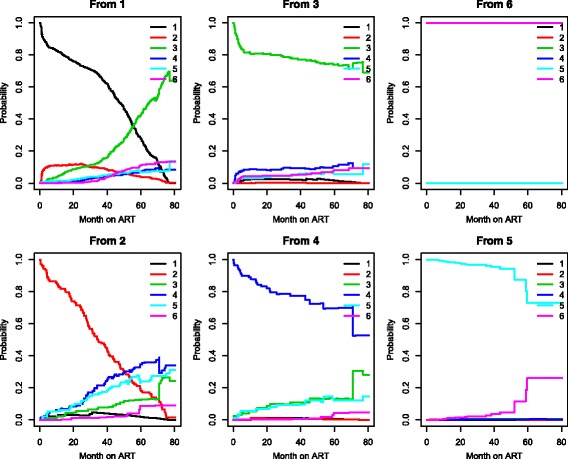
Transition probability starting from each *state*. Note: the estimate contain both direct and indirect transition probabilities. 1: d4T-3TC-NVP, 2: d4T + 3TC + EFV, 3: AZT + 3TC + NVP, 4: AZT + 3TC + EFV, 5: TDF + 3TC + EFV, 6: TDF + 3TC + NVP

The multistate model described in the previous section was estimated using the **mstate** packagee developed by [34]. Details about different R function used for the estimation is given in Additional file 1: Section S1. In particular, using the estimated transition hazards as described in Eq. (12), we calculate the transition probabilities *P*_*ℓj*_(*s*,*t*) from all starting states to all possible states, between the starting time *s*=0 and all event times successively. Note that several probabilities estimates cannot be obtained due to limited information in some states. As shown in Table 2, treatment change was observed only in 4 out of the 89 patients initiated on TDF + 3TC + NVP; hence we have chosen to consider as inadmissible the occurrence treatment modification from this treatment combination (State 6). The model has 6-states as before but with a different transition matrix. The transition matrix in Additional file 1: Section S1 shows the multistate structure which reflects this framework. We show in Fig. 3 the estimated transition probabilities from all starting states to all possible states, between the starting time *s*=0 and all event times successively. Treatment combinations containing d4T have the lowest probability of no treatment modification while the combination of TDF and EFV are the most robust to treatment modification.

**Fig. 4:**
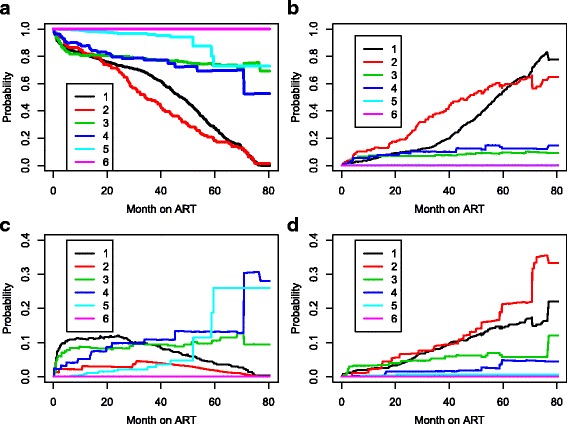
Prediction probabilities at s = 0 for a reference patient. **a** Probability of no treatment modification, **b** Probability of NRTI substitution, **c** Probability of NNRTI substitution and **d** Probability of regimen changes. 1: d4T-3TC-NVP, 2: d4T + 3TC + EFV, 3: AZT + 3TC + NVP, 4: AZT + 3TC + EFV, 5: TDF + 3TC + EFV, 6: TDF + 3TC + NVP

As mentioned above, we are mainly interested in prediction of the four measures of HAART robustness to treatment modification: (1), probability of no treatment modification, (2) NRTI substitution, (3) NNRTI substitution and (4) regimen changes. The estimated probabilities are shown in Fig. 4. As was to be expected on the basis of the previous discussion, the prospects for a patient who received the regimens containing d4T are indeed worse than those patient who received the regimens containing AZT or TDF, the former having a far larger probability of NRTI substitution, regimen changes and the lowest treatment modification-free survival probabilities.

**Fig. 5:**
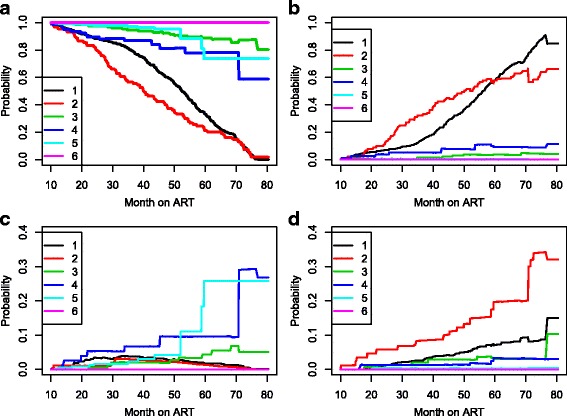
Prediction probabilities at s = 10 for a reference patient. **a** Probability of no treatment modification, **b** Probability of NRTI substitution, **c** Probability of NNRTI substitution and **d** Probability of regimen changes. 1: d4T-3TC-NVP, 2: d4T + 3TC + EFV, 3: AZT + 3TC + NVP, 4: AZT + 3TC + EFV, 5: TDF + 3TC + EFV, 6: TDF + 3TC + NVP

As mentioned above, treatment modification occurs more frequently for AZT and TDF early after treatment initiation while it occurs later on in follow-up among patients on d4T. Thus, it is interesting to compare treatments based on the situation after some months to account for early ART complication. For this, the transition probabilities at 10 months post ART were estimated and the results are presented in Fig. 5. A comparison of Figs. 4 and 5 clearly shows that the fact that a patient on a treatment combination containing AZT or TDF has not had any early ART complication leading to treatment change or modification in the first 10 months post-ART has decreased his/her probability of future treatment change or modification considerably; notably, his/her probability of long-term NRTI substitution-free survival has increased significantly. On the other hand, the long-term treatment modification-free survival of a patient on a treatment combination containing d4T was unchanged by the fact that he/she has not experienced treatment modification in the first 10 months post-ART.

### Reason for treatment modification

Table 3 shows the reason for treatment change for the total 615 observed treatment changes in the cohort, stratified by treatment combinations. We were able to obtain the reason for the majority of treatment changes (88.62%). Toxicity and comorbidity were was the main reasons for treatment modification accounting for 48.94% and 14.31% of the observed treatment changes, respectively. About 50% of the patients on all the regimens except TDF + 3TC + EFV reported toxicity or side effects. In addition, phasing out of d4T from the NRTI backbone accounts for 20.16% of the observed treatment changes.

**Table 3:** Reasons for antiretroviral modification among HIV patients on HAART

	1	2	3	4	5	6	Total
Drug out of stock	0(0.00)	0(0.00)	3(5.56)	0 (0.00)	0 (0.00)	0(0.00)	3(0.48)
Hepatitis	0(0.00)	0(0.00)	1 (1.85)	0 (0.00)	0(0.00)	0(0.00)	1(0.16)
New TB	68(16.35)	3(3.00)	15 (27.78)	0 (0.00)	1(5.88)	1(25.00)	88(14.31)
Phaseout	106(25.48)	18 (18.00)	0(0.00)	0 (0.00)	0(0.00)	0(0.00)	124(20.16)
Pregnancy	1(2.40)	6 (6.00)	0 (0.00)	5 (20.83)	11(64.70)	1(25.00)	24(3.9)
Toxicity/side effect	198(47.59)	56(56.00)	29 (53.70)	13 (54.17)	3(17.65)	2(50.00)	301(48.94)
Treatment failure	2(4.1)	1 (1.00)	1 (1.85)	0 (0.00)	0 (0.00)	0(0.00)	4(0.65)
unknown	41 (9.86)	16 (16.00)	5 (9.26)	6 (25.00)	2 (11.76)	0(0.00)	70(11.38)
Total change	416	100	54	24	17	4	615

Note: 1: d4T + 3TC + NVP, 2: d4T + 3TC + EFV, 3: AZT + 3TC + NVP, 4: AZT + 3TC + EFV, 5: TDF + 3TC + EFV, and 6: TDF + 3TC + NVP

**Fig. 6:**
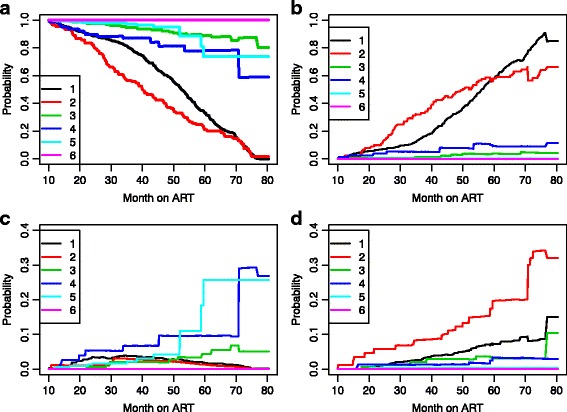
Prediction probabilities for time to treatment change due to toxicity at s = 0 for a reference patient: **a** Probability of no treatment modification, **b** Probability of NRTI substitution, **c** Probability of NNRTI substitution and **d** Probability of regimen changes. 1: d4T-3TC-NVP, 2: d4T + 3TC + EFV, 3: AZT + 3TC + NVP, 4: AZT + 3TC + EFV, 5: TDF + 3TC + EFV, 6: TDF + 3TC + NVP

In order to quantify the effect of toxicity on treatment change, we modify the definition of time-to-treatment change to time-to-treatment change due to toxicity. Here, treatment change related to other reasons during the follow-up period were censored at the time of their occurrence. As seen from Table 4, a large proportion of patients (26.25%) on NVP and d4T combination had NRTI substitution with d4T replaced by AZT due to toxicity. Similarly, 15.92% and 14.01% of patients on EFV combination with d4T had d4T replaced by AZT and TDF, respectively due to toxicity. Treatment combination of TDF and EFV was the most robust to treatment modification among the six first line regimens. As previously noted, this combination seems the least toxic. In a similar fashion, we calculate the toxicity driven transition probabilities *P*_*ℓj*_(*s*,*t*) from all starting states to all possible states, between the starting time *s*=0 and all event times successively and extract the four measures of transition probabilities. Here again, Fig. 6 shows treatment combination containing d4T had highest probability for treatment modification due to toxicity. Where as the combination of TDF and EFV was the most robust to treatment modification.

**Table 4:** Observed transition matrix

	1	2	3	4	5	6	No event	Total entering
1	-	15(2.77)	142(26.25)	2(0.36)	6(1.10)	33(6.09)	343(63.40)	541
2	2(1.27)	-	5 (3.18)	25 (15.92)	22(14.01)	1(0.63)	102(64.96)	157
3	7(1.44)	0(0.00)	-	6(1.24)	7(1.45)	9(1.85)	455(94.00)	483
4	0(0.00)	1(0.60)	5 (3.05)	-	7(4.26)	0(0.00)	151(92.07)	161
5	0(0.00)	1(0.21)	0 (0.00)	0(0.00)	-	2(0.42)	470(99.36)	471
6	0(0.00)	0(0.00)	1 (1.12)	1(1.12)	0(0.00)	-	87(97.75)	89

Note: 1: d4T + 3TC + NVP, 2: d4T + 3TC + EFV, 3: AZT + 3TC + NVP, 4: AZT + 3TC + EFV, 5: TDF + 3TC + EFV, and 6: TDF + 3TC + NVP. The *ℓ* *j* ^*th*^ entries are the frequencies (percentage) of toxicity driven transition from state *ℓ* to state *j*. Those in column "no event" are the numbers of patients ending the study in each state and those in column "total entering" are the numbers of patients observed to be in each state at some time point prior to the end of the study

## Discussion

This study has provided unique and important data on durability of first-line ART and on reasons responsible for antiretroviral treatment modification in the setting of a tertiary care Hospital in a resource limited country. This work adds to the previous observational studies [15–18, 22–27] conducted in resource-limited settings, where no distinction was made between NRTI substitution and NNRTI substitution, treatment modification due to all causes and toxicity driven treatment modification, and the incidence of subsequent treatment modification was not studied. Further, we show how a simple multi-state survival model can be used to estimate the probability of the future treatment combination of the patient given all the information available up to the present moment.

In our cohort, a large proportion of patients (41.2%) changed their treatment during follow up time, where 34.4% patients changed once and 6.69% changed more than once, which is far higher to what has been previously reported [22, 27]. The short follow up time and consideration of only the first treatment modification as event of interest in their study may be the reason for such discrepancy. Among the regimens containing d4T, 77% of 541 patients on d4T + 3TC + NVP and 64% of 157 patients on d4T + 3TC + EFV experienced all cause treatment modification. In all cause analysis, regimens containing d4T had highest probability for treatment modification, NRTI substitution, and regimen switching as compared to those regimens containing AZT and TDF, consistent with previous findings [15–18, 22–27]. Whereas the combination of TDF and EFV was the most robust to treatment modification. Apart from d4T, patients on EFV were less susceptible to treatment modification than patients on NVP, similar to what has been reported previously [27].

We also found that treatment modification occurring more frequently for AZT and TDF early after treatment initiation while treatment modification occurs later on in follow-up amongst patients on d4T, consistent with previous findings [27]. The superiority of AZT and TDF over d4T, however, should not be shadowed by this finding. A further comparison of treatment combinations accounting for early ART complication shows that, if a patient on a treatment combination containing AZT or TDF has not had any early ART complication leading to modification in the first 10 months post-ART his probability of future treatment modification decreased considerably. On the other hand, the long-term treatment modification-free survival of a patient on a treatment combination containing d4T was unchanged by the fact that he/she has not experienced treatment modification in the first 10 months post-ART. Further, no significant difference in the timing of treatment modification was observed among NVP and EFV.

The unique feature of this study is we manage to determine the type of treatment modification along with the reason for modification. Only in less than 7% of the treatment changes were we unable to determine the reason for treatment modification. Toxicity-related treatment modification has been identified as the most common reason for treatment modification accounting for 48.94% of the changes, followed by comorbidity (New TB) 14.31%, similar to what has been reported previously [15–18, 22–27]. About 50% of the patients on all the regimens except TDF plus EFV reported toxicity or side effects. The largest number of treatment modification due to toxicity was in patients on d4T: approximately 27% of those who originally started with NVP and d4T combination had NRTI substitution with d4T replaced by AZT and 15.92% and 14.01% patients who originally started with EFV combination with d4T had d4T replaced by AZT and TDF,respectively. Treatment combination of TDF and EFV was the most robust to toxicity related treatment modification among the six first line regimens. As previously noted, this combination seems the least toxic. This is a significant finding because TDF is a WHO recommended preferred treatment, with AZT as alternative [20]. Phasing out of d4T also accounted for 20.16% of treatment changes observed in our study.

This study provided unique and important data on durability of first-line ART and on reasons responsible for antiretroviral treatment modification in a resource limited setting. Furthermore, the study shows the use of multi-state models to study the evolution of patient’s state (treatment regimen) over time and to predict the probability of changing treatment. The proposed model allow us to model both the occurrence of different event types (such as, single drug substitution or regimen switch) and the occurrence of subsequent events, the latter potentially of different types in a unified way.

Our findings must be interpreted in light of some limitations. Our model assumes that the future course of a patient only depends on where you are at the current time, but not on how you got there. Deviations from this could have led to bias.

## Conclusion

Our study shows the burden of toxicity/side effect related to d4T use is a matter of major concern, as it accounts for the majority of modifications. Safer and more tolerable regimens like a combination of TDF and EFV should be made more accessible to treatment programs in resource-limited settings. Moving away from drugs with poor safety profiles, such as d4T, could reduce modification rates and this would improve regimen tolerability, while preserving future treatment options.

## Additional file

Additional file 1 Supplementary Appendix: Evaluation of the Durability of First-line Highly Active Antiretroviral Therapy in Southwest Ethiopia Using Multistate Survival Model. (PDF 218 KB)

## Abbreviations

ART: Antiretroviral therapy
HAART: Highly active antiretroviral therapy
NRTI: Nucleotide reverse transcriptase inhibitors
NNRTI: Non nucleotide reverse transcriptase inhibitors
WHO: World Health Organization
VCT: Voluntary counselling and testing
PMTCT: Prevention of mother-to-child transmission
