# Supplementary material for: Multi-state models for the analysis of time-to-treatment modification among HIV patients under highly active antiretroviral therapy in Southwest Ethiopia
Source: BMC Infect Dis. 2017 Jun 27;17:453. doi: 10.1186/s12879-017-2533-3 (PMC5488384; doi:10.1186/s12879-017-2533-3)
Supplement: Additional file 1 — Supplementary Appendix: Evaluation of the Durability of First-line Highly Active Antiretroviral Therapy in Southwest Ethiopia Using Multistate Survival Model. (PDF 218 KB) [file 12879_2017_2533_MOESM1_ESM.pdf]

positioning

RESEARCH

Supplementary Appendix: Evaluation of the Durability of First-line Highly Active Antiretroviral Therapy in Southwest Ethiopia Using Multistate Survival Model

Belay Birlie[??*], Roel Braekers[??], Tadesse Awoke[??], Adetayo Kasim[??] and Ziv Shkedy[??]

[*]Correspondence:
belaya.birlie@gmail.com
[??]Department of Statistics,
Jimma University, Jimma, Ethiopia
Full list of author information is available at the end of the article
[†]Equal contributor

S1 Data Preparation and Analysis using R

In this subsection we discuss data preparation, estimation and prediction procedures used for the analysis of the treatment history data presented in Section 2 of the main manuscript. The data used in this study are 1284 ART naive patients, aged 18 years or older and who initiated a standard, public-sector, first-line ART regimen at Jimma university specialized Hospital HIV/AIDS clinic between between January 1, 2007 and December 31, 2011. Supplementary Table S1.1 shows the treatment history of the first four patients of the cohort.

Table 1 Treatment history of the first four patients

	card.num.	Month.on.ART	Cur.ARV.regimen	regimen.change	Reason.for.change	Followup.Endtime	Reason
1	1202	0.0	1a	yes	Phaseout	78.90	study end
2	1202	75.9	1c	no		78.90	study end
3	1203	0.0	1a	yes	New TB	74.17	study end
4	1203	13.0	1b	yes	toxicity	74.17	study end
5	1203	54.6	1e	no		74.17	study end
6	1204	0.0	1a	no		7.97	Lost

Note: 1a: d4T + 3TC + NVP, 1b: d4T + 3TC + EFV, 1c: AZT + 3TC + NVP, 1d: AZT + 3TC + EFV, 1e: TDF + 3TC + EFV, and 1f: TDF + 3TC + NVP.

Patient 1204 start treatment with treatment combination 1a: d4T + 3TC + NVP and lost from follow up at 7.97 months post-ART without experiencing any event during follow up, i.e. censored at $t = 7.97$ months. Patient 1202 start therapy with treatment 1a: d4T + 3TC + NVP and changed to treatment 1c: AZT + 3TC + NVP after 75.9 months of follow up due to phasing out of d4T from NRTI list and stay on this treatment and still alive (under follow up) at 78.9 months post-ART. Patient 1203 first modify the NNRTI ($1a \to 1b$) at 13 months post-ART due to New TB, subsequently modify the NRTI ($1b \to 1e$) at 54.6 months post-ART due to toxicity, and is still alive at 74.17 months post-ART.

Our interest is to evaluate the durability of first-line treatment combinations, hence we want to model the time-to-treatment change using multistate modeling approach. The first step in a multistate model analysis is to set up the transition matrix that specifies which direct transitions are possible and which are not. We did that using the function 'transMat' from the "mstate" Package [?].

```
> tmat1 <- transMat(x = list(c(2,3,4,5,6), c(1,3,4,5,6), c(1,2,4,5,6)
+,c(1,2,3,5,6), c(1,2,3,4,6), c(1,2,3,4,5)),
+ names=c("1a", "1b", "1c", "1d", "1e", "1f"))
> tmat1
    to
from 1a 1b 1c 1d 1e 1f
  1a NA  1  2  3  4  5
  1b  6 NA  7  8  9 10
  1c 11 12 NA 13 14 15
  1d 16 17 18 NA 19 20
  1e 21 22 23 24 NA 25
  1f 26 27 28 29 30 NA
```

There are 6 initial states and 30 possible transitions for which we want to obtain probability estimates. Then, we need to prepare the data in the so so-called 'long format' that will give us more flexibility for multistate modeling modeling. The R package "mstate" [?] have a function called "msprep" for preparing data in the aforementioned format. However, we can't directly use the function msprep of the

mstate package because in our case the same *state* can be visited more than once. For this, we write our own function to construct the data set such that each subject has as many rows as transitions for which he/she is at risk (Note: The code can be available from the first author). Below we present part of the result of the new data structure.

```
treatment.change[1:30,]

     id start  stop from to status  trans   Reason
1  1202   0.0 75.90   1a 1b      0 1a->1b
2  1202   0.0 75.90   1a 1c      1 1a->1c Phaseout
3  1202   0.0 75.90   1a 1d      0 1a->1d
4  1202   0.0 75.90   1a 1e      0 1a->1e
5  1202   0.0 75.90   1a 1f      0 1a->1f
6  1202  75.9 78.90   1c 1a      0 1c->1a
7  1202  75.9 78.90   1c 1b      0 1c->1b
8  1202  75.9 78.90   1c 1d      0 1c->1d
9  1202  75.9 78.90   1c 1e      0 1c->1e
10 1202  75.9 78.90   1c 1f      0 1c->1f
11 1203   0.0 13.00   1a 1b      1 1a->1b  New TB
12 1203   0.0 13.00   1a 1c      0 1a->1c
13 1203   0.0 13.00   1a 1d      0 1a->1d
14 1203   0.0 13.00   1a 1e      0 1a->1e
15 1203   0.0 13.00   1a 1f      0 1a->1f
16 1203  13.0 54.60   1b 1a      0 1b->1a
17 1203  13.0 54.60   1b 1c      0 1b->1c
18 1203  13.0 54.60   1b 1d      0 1b->1d
19 1203  13.0 54.60   1b 1e      1 1b->1e  toxicity
20 1203  13.0 54.60   1b 1f      0 1b->1f
21 1203  54.6 74.17   1e 1a      0 1e->1a
22 1203  54.6 74.17   1e 1b      0 1e->1b
23 1203  54.6 74.17   1e 1c      0 1e->1c
24 1203  54.6 74.17   1e 1d      0 1e->1d
25 1203  54.6 74.17   1e 1f      0 1e->1f
26 1204   0.0  7.97   1a 1b      0 1a->1b
27 1204   0.0  7.97   1a 1c      0 1a->1c
28 1204   0.0  7.97   1a 1d      0 1a->1d
29 1204   0.0  7.97   1a 1e      0 1a->1e
30 1204   0.0  7.97   1a 1f      0 1a->1f
```

The data contains a patient identification column id, from and to column specifying from which state the transition initiates and to which it terminates. Furthermore, it contains a start and stop time to indicate when the patient started and stopped being at risk for that transition, and a status to denote whether or not (1 and 0, respectively) the patient reached the to state. Before we proceed to use the data for analysis, we let R to know our data set as an msdata object of class msdata

and data.frame by attaching a trans attribute holding the transition matrix defined above.

```
# creating an  msdata object
attr(treatment.change, "trans") <- tmat1
    class(treatment.change) <- c("msdata","data.frame")
```

The first important parameter that we want to estimate was the cumulative hazards and we use the function coxph() from the survival package to estimate this. This Cox model has separate baseline hazards for each of the transitions and no covariates.

```
  # Estimation
     fit1=coxph(Surv(start,stop,status)~strata(trans),
      data=treatment.change,method = "breslow")
     summary(fit1)
```

The output of coxph() is the input for mstate's function msfit(). It estimates transition hazards and their associated (co)variances. We also use the function probtrans() in order to calculate the estimated transition probabilities. As mentioned in the result section of the main manuscript, however, several probabilities estimates cannot be obtained due to limited information in some states. As shown in Table 2 of the main manuscript, treatment change was observed only in 4 out of the 89 patients initiated on TDF + 3TC +NVP; hence we have chosen to consider as inadmissible the occurrence treatment modification from this treatment combination (State 6). Hence, we setup a new transition matrix 'tmat2' with 6-states as before but with a lower possible transitions.

```
> tmat2 <- transMat(x = list(c(2,3,4,5,6), c(1,3,4,5,6), c(1,2,4,5,6)
+,c(1,2,3,5,6), c(1,2,3,4,6), c()),
+ names=c("1a", "1b", "1c", "1d", "1e", "1f"))
> tmat2
    to
from 1a 1b 1c 1d 1e 1f
  1a NA  1  2  3  4  5
  1b  6 NA  7  8  9 10
  1c 11 12 NA 13 14 15
  1d 16 17 18 NA 19 20
  1e 21 22 23 24 NA 25
  1f NA NA NA NA NA NA
```

Then, we modify the trans attribute holding the transition matrix of our msdata, refit the stratified cox model and obtain estimated transition probabilities using msfit and probtrans function of msdata.

```
   # Prediction
     msf0 <- msfit(object=fit2, trans=tmat2)

     pt0 <- probtrans(msf0, predt = 0,direction="forward",method = "greenwood")
     pt0 <- probtrans(msf0, predt = 10,direction="forward",method = "greenwood")
```

The argument predt in the probtrans function gives the starting time for prediction and the "forward" option in the argument direction means that the prediction is made from predt. That is, the starting time s for the calculation of the transition probabilities in $P(s;t)$ remains fixed at the value predt, while time t varies from s to the last (possibly censored) time point in the data.

S2 Transition intensities and Transition Probabilities

In this study we used a Six-state multistate model for describing treatment treatment history of patients. The two important parameters for describing treatment history of patients are the transition intensities matrix, $\mathbf{A}(t)$, and the transition probability matrix, $\mathbf{M}(s,t)$. The off-diagonal (ℓ, j) elements of $\mathbf{A}(t)$ denote the hazard of making $\ell \rightarrow j$ transition and the diagonal elements are defined as minus the sum of the transition intensities of the transitions out from state ℓ. Similarly, the off-diagonal (ℓ, j) elements of $\mathbf{M}(s,t)$ denote the transition probability from state ℓ to state j in the time interval $(s, t]$.

$$\mathbf{A}(t) = \begin{pmatrix} A_{11}(t) & A_{12}(t) & A_{13}(t) & A_{14}(t) & A_{15}(t) & A_{16}(t) \\ A_{21}(t) & A_{22}(t) & A_{23}(t) & A_{24}(t) & A_{25}(t) & A_{26}(t) \\ A_{31}(t) & A_{32}(t) & A_{33}(t) & A_{34}(t) & A_{35}(t) & A_{36}(t) \\ A_{41}(t) & A_{42}(t) & A_{43}(t) & A_{44}(t) & A_{45}(t) & A_{46}(t) \\ A_{51}(t) & A_{52}(t) & A_{53}(t) & A_{54}(t) & A_{55}(t) & A_{56}(t) \\ A_{61}(t) & A_{62}(t) & A_{63}(t) & A_{64}(t) & A_{65}(t) & A_{66}(t) \end{pmatrix}$$

$$\mathbf{M}(s,t) = \begin{pmatrix} P_{11}(s,t) & P_{12}(s,t) & P_{13}(s,t) & P_{14}(s,t) & P_{15}(s,t) & P_{16}(s,t) \\ P_{21}(s,t) & P_{22}(s,t) & P_{23}(s,t) & P_{24}(s,t) & P_{25}(s,t) & P_{26}(s,t) \\ P_{31}(s,t) & P_{32}(s,t) & P_{33}(s,t) & P_{34}(s,t) & P_{35}(s,t) & P_{36}(s,t) \\ P_{41}(s,t) & P_{42}(s,t) & P_{43}(s,t) & P_{44}(s,t) & P_{45}(s,t) & P_{46}(s,t) \\ P_{51}(s,t) & P_{52}(s,t) & P_{53}(s,t) & P_{54}(s,t) & P_{55}(s,t) & P_{56}(s,t) \\ P_{61}(s,t) & P_{62}(s,t) & P_{63}(s,t) & P_{64}(s,t) & P_{65}(s,t) & P_{66}(s,t) \end{pmatrix}$$

For Markov models there is a powerful relation between these transition probabilities and the transition intensities [**?**], given by

$$\mathbf{M}(s,t) = \prod_{u \in (s,t]} (\mathbf{I} + d\mathbf{A}(u))$$

$$= \prod_{u \in (s,t]} \left\{ \begin{pmatrix} 1 & 0 & 0 & 0 & 0 & 0 \\ 0 & 1 & 0 & 0 & 0 & 0 \\ 0 & 0 & 1 & 0 & 0 & 0 \\ 0 & 0 & 0 & 1 & 0 & 0 \\ 0 & 0 & 0 & 0 & 1 & 0 \\ 0 & 0 & 0 & 0 & 0 & 1 \end{pmatrix} + d \begin{pmatrix} -A_{1.}(u) & A_{12}(u) & A_{13}(u) & A_{14}(u) & A_{15}(u) & A_{16}(u) \\ A_{21}(u) & -A_{2.}(u) & A_{23}(u) & A_{24}(u) & A_{25}(u) & A_{26}(u) \\ A_{31}(u) & A_{32}(u) & -A_{3.}(u) & A_{34}(u) & A_{35}(u) & A_{36}(u) \\ A_{41}(u) & A_{42}(u) & A_{43}(u) & -A_{4.}(u) & A_{45}(u) & A_{46}(u) \\ A_{51}(u) & A_{52}(u) & A_{53}(u) & A_{54}(u) & -A_{5.}(u) & A_{56}(u) \\ A_{61}(u) & A_{62}(u) & A_{63}(u) & A_{64}(u) & A_{65}(u) & -A_{6.}(u) \end{pmatrix} \right\}$$

More precisely, $P_{12}(s,t) = P(X_t = 2 \mid X_s = 1), \quad s \leq t, u \in [0,s]$ is the entry $(1,2)$ of the solution of the above equation. Entry $(1,3)$ is $P(X_t = 3 \mid X_s = 1), \quad s \leq t, u \in [0,s]$, and the probability of staying in treatment 1, $P_{11}(s,t) = P(X_t = 1 \mid X_s = 1)$, is in entry $(1,1)$.

S3 Duration in treatment combination before change

Figure S3.1 presents the waiting time in original treatment before switch. We summarize the waiting time in the specified treatment combination only for patients who changed their treatment.

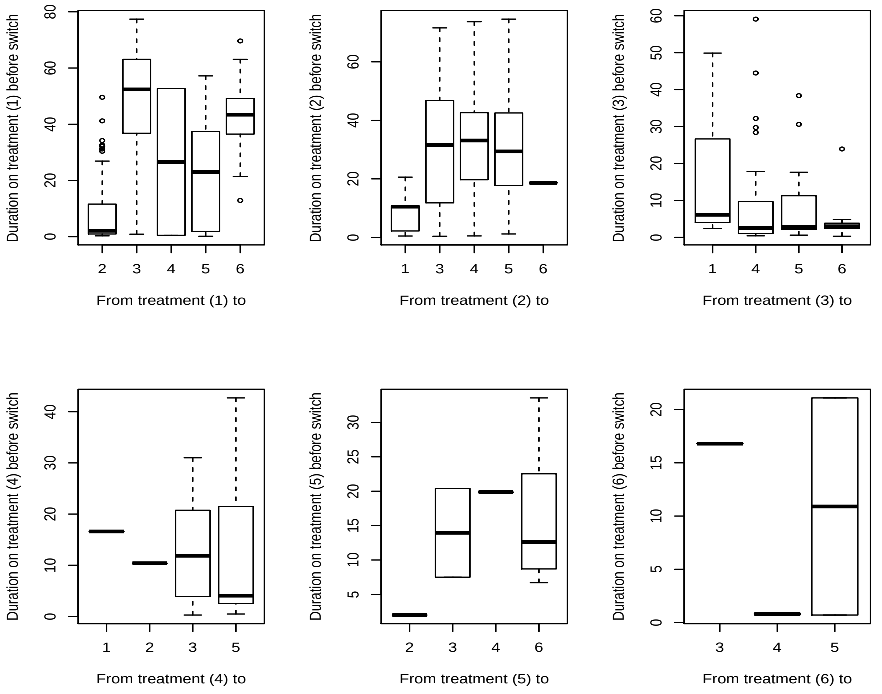

Figure S3.1. Time elapsed in original treatment combinations before switch

[xscale=6, yscale=8,¿=stealth] v=[circle, minimum size=1mm,draw,thick] [v] (a) 1; [v] (b) [right=of a] 2; [v] (c) [below=of a] 2; [v] (d) [below=of b] 1; [thick,-¿] (a) to node (c); [thick,-¿] (a) to node (d); [thick,-¿] (b) to node (d);
